# Brazilian *Anopheles darlingi* Root (Diptera: Culicidae) Clusters by Major Biogeographical Region

**DOI:** 10.1371/journal.pone.0130773

**Published:** 2015-07-14

**Authors:** Kevin J. Emerson, Jan E. Conn, Eduardo S. Bergo, Melissa A. Randel, Maria Anice M. Sallum

**Affiliations:** 1 Biology Department, St. Mary’s College of Maryland, St. Mary’s City, Maryland, United States of America; 2 The Wadsworth Center, New York State Department of Health, Albany, New York, United States of America; 3 Department of Biomedical Sciences—School of Public Health, SUNY Albany, Albany, New York, United States of America; 4 Superintendência de Controle de Endemias, Secretaria de Estado da Saúde de São Paulo, Araraquara, São Paulo, Brazil; 5 Institute of Molecular Biology, University of Oregon, Eugene, Oregon, United States of America; 6 Departamento de Epidemiologia, Faculdade de Saúde Pública, Universidade de São Paulo, São Paulo, Brazil; Centro de Pesquisas René Rachou, BRAZIL

## Abstract

The major drivers of the extensive biodiversity of the Neotropics are proposed to be geological and tectonic events together with Pliocene and Pleistocene environmental and climatic change. Geographical barriers represented by the rivers Amazonas/Solimões, the Andes and the coastal mountain ranges in eastern Brazil have been hypothesized to lead to diversification within the primary malaria vector, *Anopheles *(*Nyssorhynchus*) *darlingi* Root, which primarily inhabits rainforest. To test this biogeographical hypothesis, we analyzed 786 single nucleotide polymorphisms (SNPs) in 12 populations of *An*. *darlingi* from across the complex Brazilian landscape. Both model-based (STRUCTURE) and non-model-based (Principal Components and Discriminant Analysis) analysis of population structure detected three major genetic clusters that correspond with newly described Neotropical biogeographical regions: 1) Atlantic Forest province (= southeast population); 2) Parana Forest province (= West Atlantic forest population, with one Chacoan population - SP); and 3) Brazilian dominion population (= Amazonian population with one Chacoan population - TO). Significant levels of pairwise genetic divergences were found among the three clusters, allele sharing among clusters was negligible, and geographical distance did not contribute to differentiation. We infer that the Atlantic forest coastal mountain range limited dispersal between the Atlantic Forest province and the Parana Forest province populations, and that the large, diagonal open vegetation region of the Chacoan dominion dramatically reduced dispersal between the Parana and Brazilian dominion populations. We hypothesize that the three genetic clusters may represent three putative species.

## Introduction

*Anopheles* (*Nyssorhynchus*) *darlingi* Root is broadly distributed in Central and South America, extending from southeastern Mexico to northern Argentina and from east of the Andes to the Atlantic coast [1]. This species is the most aggressive and effective Neotropical malaria vector, primarily in the Amazon/Solimões River basin. Furthermore, *An*. *darlingi* is associated with malaria dynamics in forest areas where the natural ecosystems are undergoing intensive ecological changes promoted by deforestation and land use [2, 3].

*Anopheles darlingi* was described by Root [4] based on morphological characters of the egg, fourth-instar larva, pupa, male and female collected in Caxiribú in the vicinity of Porto das Caixas, Rio de Janeiro state, Brazil. Galvão et al. [5] expanded the geographical distribution of the species to inland São Paulo state, Bahia, and northern Brazil. *Anopheles paulistensis* Galvão, Lane and Corrêa was described as a morphological variant of *An*. *darlingi* based on differences in the egg, male and female morphology of specimens from Pereira Barreto, inland São Paulo state and Manaus, Amazonas state [5]. Later, Lane [6] considered that those differences represented phenotypic variations, and *An*. *paulistensis* was synonymized with *An*. *darlingi*. Polymorphisms were also observed in the banding pattern of the X and all four autosome arms of the salivary gland polytene chromosome of representatives of *An*. *darlingi* populations from three northern localities in the Amazon forest and one southern locality in the domain of Cerrado, inland São Paulo state, and considered to be linked with distinct vectorial capacity [7]. More recently, Malafronte et al. [8] observed intraspecific variability in the rDNA ITS2 sequences that corroborated the northern / southern population polymorphisms in the polytene chromosomes detected by Kreutzer et al. [7]. Furthermore, heterogeneities were also observed in the peak biting behavior [9, 10], in wing morphometric geometry [11], in vectorial capacity [12], and in the genetic structure of southeastern and northern populations using both mtDNA Cytochrome Oxidase I (*COI*) [13], and microsatellite markers [14]. In contrast, *An*. *darlingi* has been considered to be a monotypic species based on other data sets [15, 16].

Using specimens spanning almost the entire distribution of *An*. *darlingi*, *COI* sequences [17] and microsatellite loci [18] detected deep geographic differentiation that separates Amazonian South America populations from those in Central America, northwestern Colombia and Venezuela. Ancient evolutionary processes were invoked to explain the *COI* split [17]; in contrast, distance and differences in effective population sizes best explained the level of differentiation detected by microsatellites [18].

Within South American populations, variation in *COI* resolved two genetic clusters that coincide with two centers of endemism: 1) within the Amazonas/Solimões river basin plus Guyana (north of the Amazon), and 2) within South America (Belém, Pará), with expansions that occurred during the Pleistocene [17]. Subsequently, it was found that the population growth of *An*. *darlingi* was not homogeneous [13]. Geographical barriers represented by the rivers Amazonas/Solimões, the Andes, and the coastal mountain ranges in eastern Brazil resulted in at least four subgroups within the South American cluster [13]. It is worthwhile noting that the populations from the lowlands along the Atlantic coast in Rio de Janeiro and Espírito Santo states were markedly distinct from those of central Amazonia, southern and northeast Brazil.

The Atlantic Forest, originally approximately 150 million hectares, is one of the largest tropical rainforests in the Americas. Its extreme latitudinal dimension (about 29 degrees) and an altitudinal span from sea level (Atlantic coast) to ~2800m (Serra do Mar and Serra da Mantiqueira), incorporates tropical and subtropical zones with diverse environmental conditions [19]. The variable landscape, ecology and terrain favor high biological diversity and multiple areas of plant and animal endemism [20, 21]. In this context, Pedro and Sallum [13] demonstrated that populations of *An*. *darlingi* from the southeastern and inland Atlantic Forest differ substantially, and hypothesized that the major geographic barrier represented by the coastal mountain range limited the dispersal of populations across the Atlantic Forest.

The Neotropical region consists mainly of forest biomes, with some extensive open vegetation biomes along a wide diagonal that comprises the Pampa, Chaco, Cerrado and Caatinga provinces [22]. Gradual development of this open vegetation promoted the separation of one former region into two: 1) northwestern South America and Amazonian forests; and 2) Parana and Atlantic forests [23]. Based on results of a rigorous cladistic biogeographical analysis of 30 plant and animal taxa, Morrone [22] proposed a system of natural sub-regions and dominions, provinces and districts, which have been categorized into hierarchical levels linked to major tectonic and geological events. At least some of the differentiation observed in *An*. *darlingi* populations may be attributed to biogeographical events that delineated the Neotropical region. We hypothesize that the development of the open vegetation area comprising the Chacoan dominion, also known as the Chaco, Cerrado and Caatinga biomes, is one of the primary isolating mechanisms that promoted the genetic differentiation of *An*. *darlingi* population groups (central Amazonia, southern Brazil and southeastern Brazil) proposed by Pedro and Sallum [13].

Herein, we use genotyping by sequencing with nextRAD (nextera-tagmented, Reductively Amplified DNA) markers (Etter et al, paper in preparation) to detect SNPs, which increase marker-resolution approximately three orders of magnitude compared with previous population genetic studies in *An*. *darlingi* [8, 13–15, 17, 18, 24, 25]. We propose to: 1) assess the level of structure among populations of *An*. *darlingi* throughout Brazil; 2) address how genetic diversity is distributed between and within the major forest domains of Amazonia and Atlantic Forest compared with Cerrado; 3) examine whether divergence among population subgroups from the Atlantic coast and central Amazonia, southern and northeast Brazil [13], are consistent with the early morphological division proposed between the variant *An*. *paulistensis* and *An*. *darlingi*; 4) address the hypothesis that the Amazonian population represents an unknown putative species; and 5) discuss patterns of structure in the context of Neotropical biogeographical regionalization [26].

## Materials and Methods

### Field Mosquito Sampling Strategy

**Fig 1:**
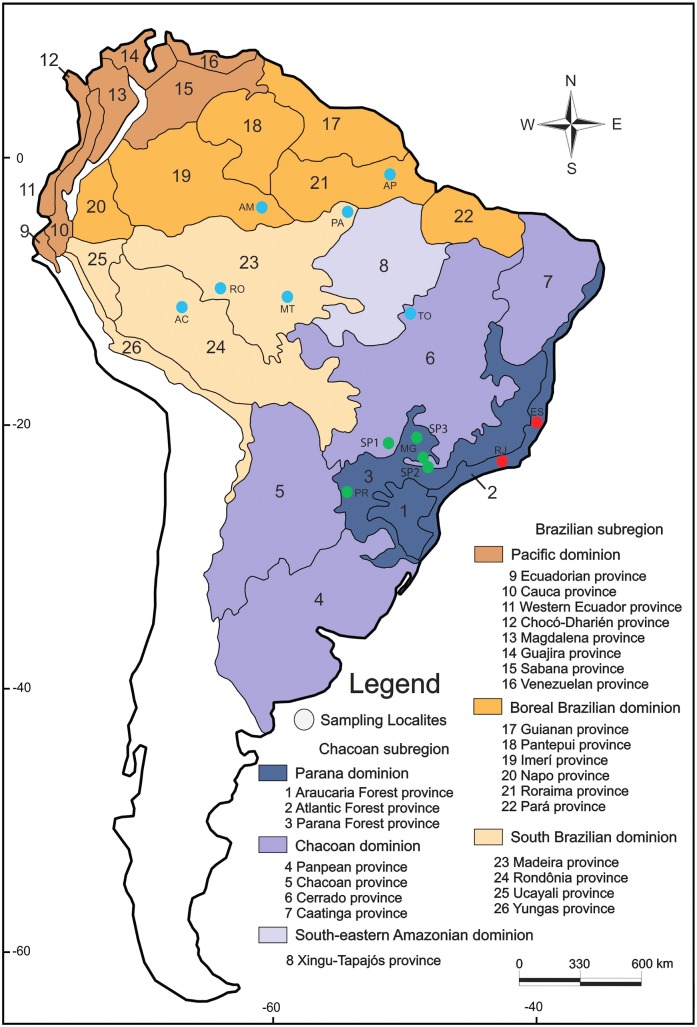
Collection sites of *Anopheles darlingi* in relation to biogeographical classification of the Neotropical region proposed by Morrone [26] Colored (blue, red and green) circles represent the inferred genetic clusters provided by results of STRUCTURE analysis. The two capital letters outside the circles represent the state where the sampling collections were carried out. AM = Amazonas; AP = Amapá; AC = Acre; RO = Rondônia; MT = Mato Grosso; PA = Para; TO = Tocantins; SP = São Paulo; MG = Minas Gerais; PR = Paraná; RJ = Rio de Janeiro; ES = Espírito Santo. The map is modified from Morrone [26].

Specimens of *An*. *darlingi* were chosen from field collections in twelve states in Brazil (Table 1) to represent two major subregions proposed by Morrone [2]: 1) Brazilian subregion (AC, AM, AP, MT, PA, RO), and 2) Chacoan subregion (ES, MG, PR, RJ, SP, TO) (Fig 1, Table 2). Populations from the Chacoan subregion were subdivided into Parana dominion, which includes the Parana Forest province, here named West Atlantic Forest population (MG, PR, and the two more southern SP sampling localities; Fig 1) and the Atlantic Forest province, here designated as southeast population (ES, RJ). In addition, sampling from the Chacoan subregion included representatives from the Cerrado province (the northwestern SP sample locality, TO) of the Chacoan dominion. Individuals of the Brazilian subregion were from the South Brazilian dominion (AC, MT, PA, RO) and the Boreal Brazilian dominion (AM, AP) (Fig 1), here named Amazonian population.

**Table 1:** Sampling localities information and their respective geographical coordinates by state in Brazil.

State	State code	Collection date	Latitude	Longitude	# Individuals
Acre	AC	July 2006	-10.1233	-66.9107	6
Amapá	AP	July 2006	-0.2131	-50.9722	5
Amazonas	AM	Feb 2009	-2.6208	-60.9439	3
Espírito Santo	ES	Oct 2007	-19.0834	-39.8844	4
Minas Gerais	MG	Nov 2006	-20.0021	-49.0795	3
Pará	PA	Oct 2008	-2.7465	-54.227	5
Paraná	PR	May 2007	-24.2715	-54.2906	6
Rio de Janeiro	RJ	May/June 2007	-22.6333	-42.3	6
Rondônia	RO	Jan 2008	-8.7667	-63.9	2
Mato Grosso	MT	May 2007	-9.4108	-59.0228	2
São Paulo*	SP1	Apr 2012	-20.5572	-51.0152	4
São Paulo*	SP2	May 2009	-22.1347	-48.3917	1
São Paulo*	SP3	May 2009	-22.0757	-48.4374	4
Tocantins	TO	Jul 2009	-10.5859	-49.6898	6

*There were no significant differences among the samples from the 3 localities in São Paulo state, so they were combined for all analyses. SP1 is the type locality of *An*. *paulistensis*, which is the farthest to the north and west of the three SP localities.

**Table 2:** Sampled populations, including the inferred genetic clusters, subdivided into biogeographical subregions, dominions and provinces proposed by Morrone [26].

State	State code	Subregion	Dominion	Province	Inferred cluster
Amapá	AP	Brazilian	Boreal Brazilian	Roraima	3
Amazonas	AM	Brazilian	Boreal Brazilian	Imerí	3
Acre	AC	Brazilian	South Brazilian	Rondônia	3
Mato Grosso	RO	Brazilian	South Brazilian	Madeira	3
Rondônia	MT	Brazilian	South Brazilian	Madeira	3
Pará	PA	Brazilian	South Brazilian	Madeira	3
Tocantins	TO	Chacoan	Chacoan	Cerrado	3
São Paulo	SP1	Chacoan	Chacoan	Cerrado	2
Minas Gerais	MG	Chacoan	Parana	Parana Forest	2
Paraná	PR	Chacoan	Parana	Parana Forest	2
São Paulo	SP2, 3	Chacoan	Parana	Parana Forest	2
Espírito Santo	ES	Chacoan	Parana	Atlantic Forest	1
Rio de Janeiro	RJ	Chacoan	Parana	Atlantic Forest	1

All necessary permits were obtained for the described field studies. Collections were made under per- manent permit number 16938–1 from Instituto Brasileiro do Meio Ambiente e dos Recursos Naturais Renováveis (IBAMA) to Maria Anice M. Sallum and E. S. Bergo. Specific permission was not required for these loca- tions as permission to collect was granted under the permanent permit. The collection locations were not privately owned or protected in any way. The field studies did not involve protected or endangered species.

Mosquitoes were captured either as larvae/pupae or adults. Males and females were collected using Shannon traps. Both adults and immature stages were sampled from multiple habitat types, such as riverside, lakeside, large farm, natural reserve and agricultural settlement, to maximize within region heterogeneity and to reduce the risk of collecting related individuals, particularly in larval habitat.

### DNA Extraction and Modified Nextera DNA Sample Preparation

Genomic DNA was extracted (Qiagen DNAEasy kit) from 57 individual mosquitoes (S1 Table) representing 12 populations (SP1, SP2 and SP3 are a single population; Table 1). The DNA was then dried, stored, and later prepared following nextRAD protocols. The nextRAD method uses a selective PCR primer to amplify genomic loci consistently between samples. Genomic DNA (7.5 ng) was first fragmented using a 1/10th Nextera reaction (Illumina, Inc), which also ligates short adapter sequences to the ends of the fragments. Fragmented DNA was then amplified using Phusion Hot Start Flex DNA Polymerase (NEB), with one of the Nextera primers modified to extend 8 nucleotides into the genomic DNA with the selective sequence TGCAGGAG. Thus, only fragments starting with a sequence that can be hybridized by the selective sequence of the primer were efficiently amplified. The following PCR parameters were used: 72°C for 3 minutes, 98°C for 3 minutes, 24 cycles of 98°C for 45 seconds followed by 75°C for 1 minute, then hold at 4°C. The dual-indexed samples were pooled and the resulting library was purified using Agencourt AMPure XP beads at 0.75 X. The purified library was then size selected to 350–500 base pairs. Sequencing was performed in 101-cycles in one lane of an Illumina HiSeq2000 (Genomics Core Facility, University of Oregon).

### STACKS and Population Genetic Analyses

Raw Illumina sequences (NCBI SRA Accession numbers SRS950393-SRS950449) were processed with STACKS v1 [27, 28]. Briefly, the raw sequences were quality-filtered using the STACKS program process_radtags. Each of the quality-filtered reads was mapped to the *An*. *darlingi* genome using bowtie [29]. The reference-genome mapped sequences were then analyzed with STACKS program ref_map.pl. Genotype assignments were corrected using the automated correction module rxstacks. A single SNP position from each RAD locus that had a minimum allele depth of 5 sequences and was scored in at least 50% of individuals within a population was retained and all of these SNP positions used for STRUCTURE analysis [30] for K values between 1 and 8, with 20–40 replicates for each K value. This analysis used a custom script that allows for parallel processing of STRUCTURE analyses (genome.smcm.edu/emersonLab/software). STRUCTURE was run with the admixture model and correlated allele frequencies, and each run used a burnin of 100,000 generations and ran an MCMC chain of 1,000,000 generations. To determine the optimal value of *K* for our samples, we used the Evanno method [31] implemented in structureHarvester [32]. A complete bash script outlining the parameters used for each component of the STACKS pipeline is provided (S1 Text). Further analysis used a limited SNP dataset that included only those loci (n = 786) that were genotyped in > 75% of individuals in each of the three clusters determined by the full SNP dataset STRUCTURE results. Principle Components Analysis was performed using the R package SNPRelate [33] and AMOVA analysis was performed using Arlequin 3.5 [34].

Due to the possibility of bias introduced in model-based (i.e., STRUCTURE) analyses, particularly due to relatively low numbers of sequences at each locus, we also implemented a Discriminant Analysis of Principal Components (DAPC) [35], implemented in the R package adegenet [36], that does not make any assumptions about the underlying population genetic models. The number of clusters inferred was determined by 100 replicate iterations of K-means clustering using the find.clusters algorithm in adegenet [36].

## Results

### NextRAD genotyping

An average of 1,625,745 (range: 229,304–5,965,810) 101bp, Illumina reads were aligned to the *An*. *darlingi* reference genome [37] and resulted in genotype calls at 18,027 (+/- 7,469 SD) loci per individual. Within individuals, 10.83% +/- 0.37 SE loci were heterozygous. Initial filtering of the SNP dataset to include only loci that were genotyped in a majority of individuals from at least one geographical region resulted in a total of 11,533 loci (S1 Table).

### Clustering of individuals

There is no evidence of isolation-by-distance among the 12 populations surveyed (Mantel test: r = 0.02, P = 0.36) that cover a range of 219 to 3,059 km. Therefore we used STRUCTURE [30], Principal Components Analysis, and Discriminant Analysis of Principal Components (DAPC) to further dissect levels of population structure [38].

Based on 11,553 loci, Bayesian clustering analysis via STRUCTURE supports three genetic clusters of *An*. *darlingi* in Brazil (S1 Fig): (1) cluster 1 consists of individuals from Atlantic Forest province (= southeast) populations (ES and RJ), (2) cluster 2 consists of Parana Forest province, with one Chacoan dominion population (= West Atlantic forest) (PR, SP, MG), and (3) cluster 3 consists of Brazilian dominion, with one Chacoan dominion population (= Amazonian) (AM, AC, AP, MT, PA, RO, TO).

### Filtering of the SNP dataset

**Fig 2:**
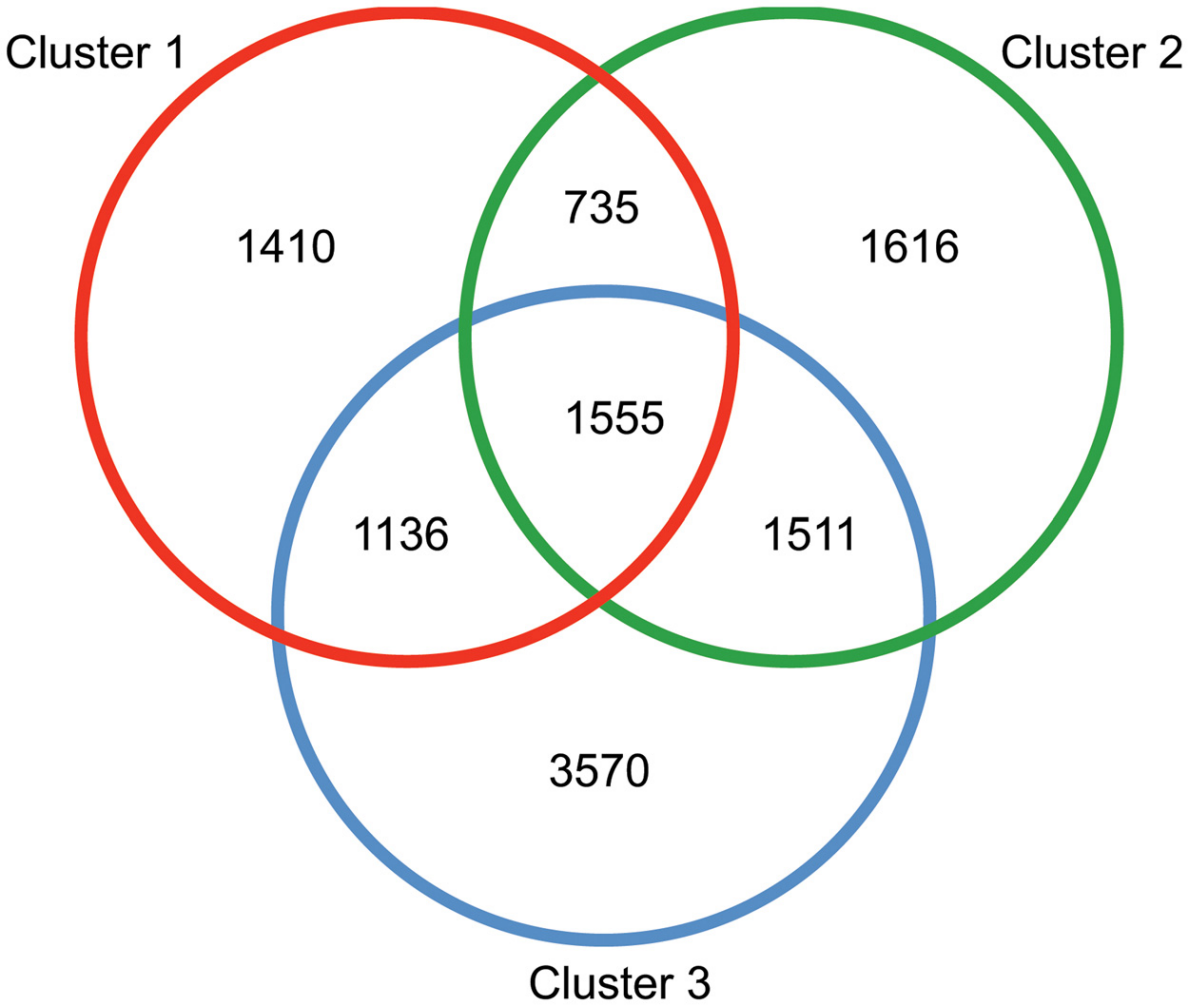
Venn diagram showing the number of private and shared genotyped loci of *An*. *darlingi*, based on loci that were genotyped in at least 50% of individuals from each cluster. The Amazonian populations (cluster 3) has the largest number of private *loci*. Of the 1,555 *loci* shared among all clusters, 786 were genotyped in at least 75% of individuals in each cluster and were included in the final, filtered SNP dataset.

Once this initial level of population structure was assessed, the genotype dataset was further filtered in order to minimize the possible bias on population genetic inferences due to missing genotype data [39]. The majority of loci genotyped were only scored in one or two of the three genetic clusters (Fig 2). Of the 11,533 loci for which genotypes were reliable inferred 1,555 loci were genotyped in individuals from all three clusters and 786 loci were genotyped in > 75% of individuals in each of the three genetic clusters. This filtered dataset of 786 loci was used for downstream analysis.

### Population Genetic Inference

**Fig 3:**
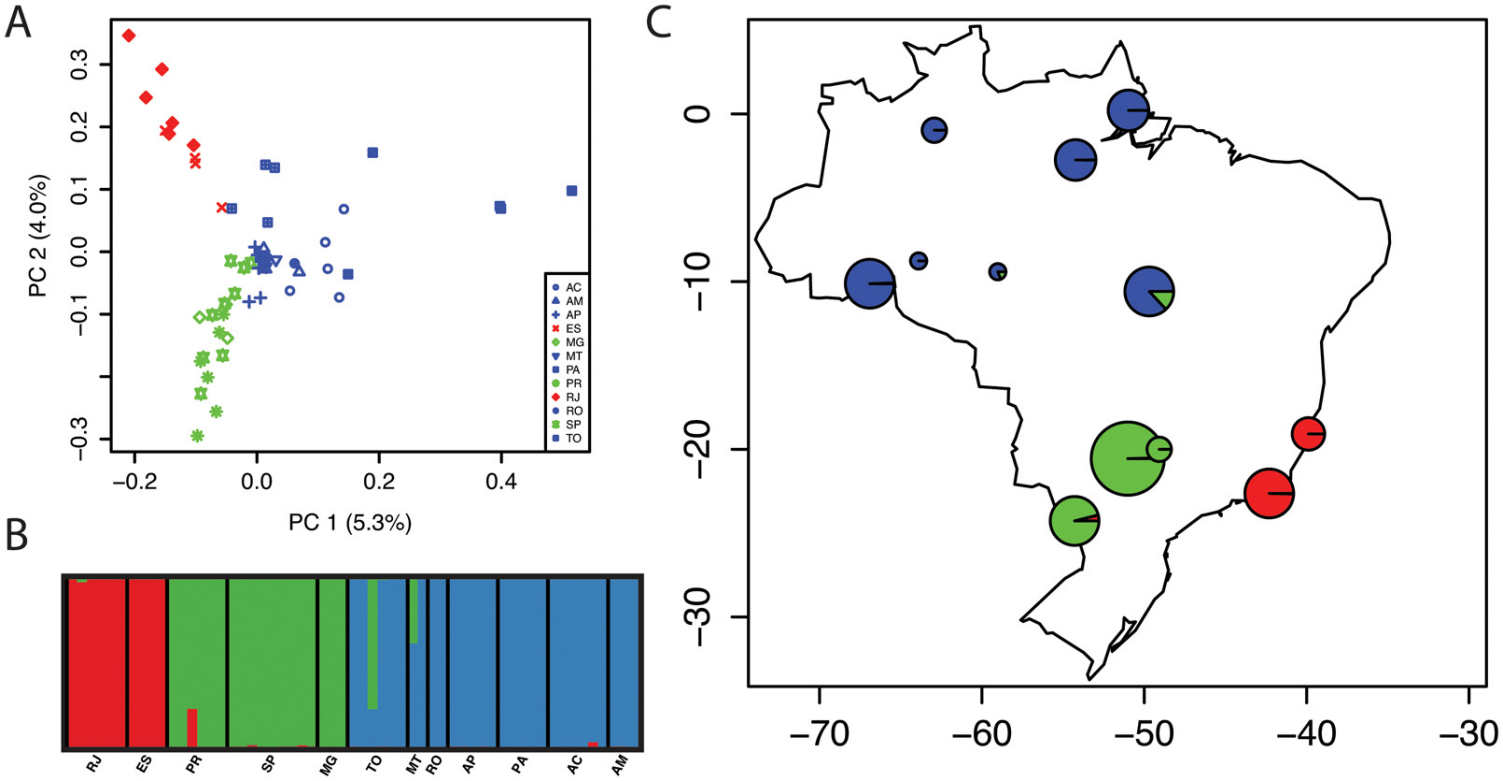
Results of Principal Components Analysis (PCA) and STRUCTURE analysis of *Anopheles darlingi* populations using the filtered SNP dataset (786 loci). (A) PCA of all loci that were shared among all three clusters (B) Results of STRUCTURE analysis depicting three inferred genetic clusters. (C) Map of collection sites showing the relative admixture of the populations. Colors reflect cluster assignment: cluster 1, red; cluster 2, green; cluster 3, blue, and the size of the pie chart is a function of the number of individuals genotyped from that population.

STRUCTURE analysis of the filtered SNP dataset discriminated three distinct genetic clusters as outlined above (Fig 3B and 3C). There were very low levels of allele sharing present, with one individual from cluster 2 showing mixing with cluster 1, and two individuals from cluster 3 showing mixing with cluster 2 (S2 Table).

Principal Components Analysis (PCA) showed clear partitioning of the populations in the first two principal components (Fig 3A). The first principal component (PCA1 5.3%) clearly discriminated the Amazonian (cluster 3) and non-Amazonian (clusters 1 and 2) populations, and the second principal component (PCA2: 4.0%) discriminated the non-Amazonian populations. Coefficients of inbreeding were all not significantly different than zero (Table 3).

**Table 3:** Summary statistics for the three inferred clusters of *Anopheles darlingi*.

			Average number of	Genome-wide	
Cluster	Description	N	individuals per locus	Pi ± SE	Fis
1	Southeast	10	7.6	0.0766 ± 0.0477	-0.0498^NS^
2	West Atlantic	18	14.4	0.0923 ± 0.0611	-0.0396^NS^
3	Amazon	29	22.4	0.1296 ± 0.0765	-0.0134^NS^

^NS^ Not Significant

**Fig 4:**
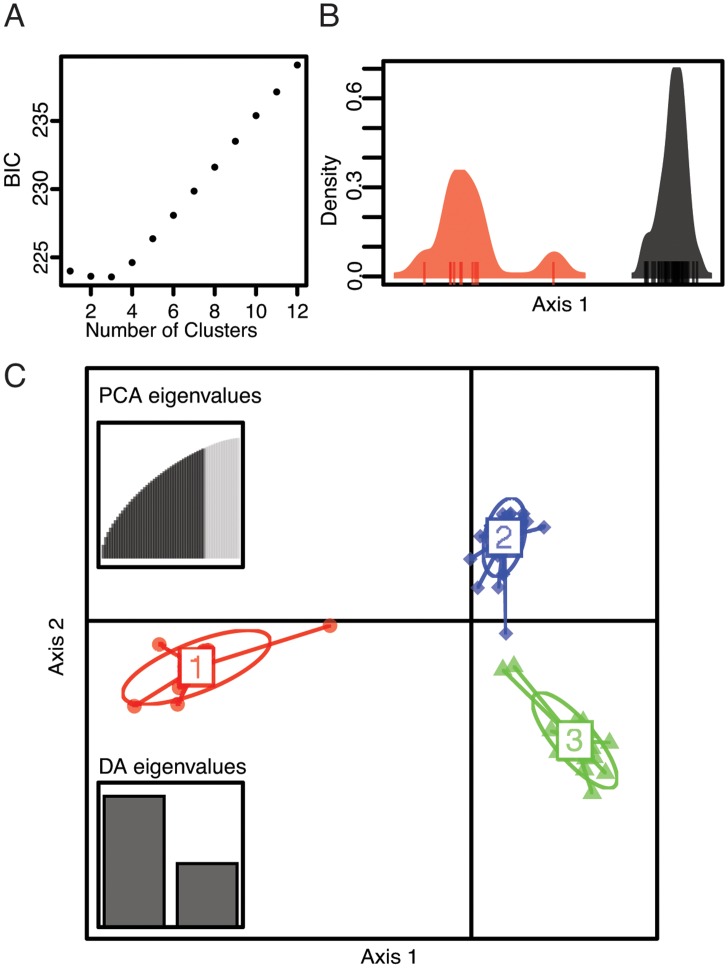
Summary of the discriminant analysis of principal components (DAPC). (A) Mean values of Bayesian Information Criterion (BIC) values for each of the values considered for K-means clustering. (B) Ordination for two clusters that separates the Atlantic Forest populations from all others along a single axis. (C) Ordination for three clusters that separate the Atlantic Forest (red, cluster 1), Parana Forest (green, cluster 2), and Amazon (blue, cluster 3) populations. The insets show the distribution of eigenvalues for the PCA and for the DAPC.

In the DAPC analysis, there was no clear ‘best’ value for the number of clusters, with the Bayesian Information Criterion (BIC) value for one, two, or three clusters, being very similar (Fig 4A). Therefore we consider both the case where there are 2 (Fig 4B) and 3 (Fig 4C) clusters. If genotypes are partitioned in to two distinct clusters, there is a clear delineation of the Atlantic Forest populations (cluster 1 above) from the Amazon and Parana Forest populations (clusters 2 and 3 above) (Fig 4B). If we partition our genotypes in to three distinct clusters, the clusters are identical to those from the STRUCTURE analysis. We assessed the robustness of these results by performing one hundred replicate analyses using the algorithm find.clusters (from adegenet [36]) for each of the above clustering schemes and individuals were always placed in to the same clusters.

There were significant levels of pairwise genetic divergence among the three clusters (AMOVA, overall F_st_ = 0.20, P < 0.001) with the highest genome-wide divergence between the southeast and West Atlantic populations: southeast population—West Atlantic population (Cluster 1 –Cluster 2; F_st_ = 0.11, P < 0.01), southeast population—Amazon population (Cluster 1 –Cluster 3; F_st_ = 0.06, P < 0.01), and West Atlantic population—Amazon population (Cluster 2 –Cluster 3; F_st_ = 0.06, P < 0.01). There was also significant level of genetic divergence between the multiple Amazonian populations as compared with the non-Amazonian populations (F_st_ = 0.05, P < 0.01).

## Discussion

Reduced representation genomic library methods, including nextRAD, suffer from sampling biases as there are usually large numbers of loci that are genotyped in only one or a few individuals [40]. Simulations have shown that datasets that are filtered to minimize the amount of missing data are more likely to accurately reflect population genetic inferences [39]. Under such filtering schemes, loci that are more highly divergent among samples tend to be excluded from the filtered datasets and thus any derived estimates of divergence are likely underestimates of true divergence values. In the data presented here, of the ~11,000 loci that were reliably genotyped in more than 50% of individuals in at least one cluster, only 768 loci were genotyped in more than 75% of individuals in all clusters. The smaller, filtered dataset was used for the majority of analyses to minimize the impact of bias due to the genotype sampling.

Support for geographical differentiation in *An*. *darlingi* depends on the markers scored and the locations sampled, similar to results in other mosquitoes (e.g. [41, 42]). For single-locus *COI* gene sequences, Mirabello & Conn [17], studying sampling locations spanning distances from 2–4,870 km, detected the highest levels of genetic differentiation between Central America and northern Amazonia, even though specimens from São Paulo and Mato Grosso states, both south of the Amazon River, were included in the analysis. Within the Brazilian Amazon [14, 25] and between Central and South America [18], microsatellite markers detected highly significant geographic differentiation. Pedro and Sallum [13], by including individuals representing the Atlantic Forest and Parana Forest provinces of the Parana dominion, Chacoan subregion, found strong evidence of population splits that are primarily coincident with the Chacoan and Brazilian subregions proposed by Morrone [26]. Even though microgeographic differentiation was not detected between neighboring Colombian states [43], Angêlla et al. [24] identified two genetically distinct sub-populations adapted to different seasonal and climatic conditions in localities along the Madeira River, Rondônia state, Brazil. Taken together these studies imply that Neotropical landscape barriers are primary drivers of divergence in *An*. *darlingi* at regional and continental scales, and that distance and environmental conditions contribute to differentiation at a local scale.

Several approaches were employed in the present study to address genomic variation among *An*. *darlingi* populations and to test whether clusters are consistent with well-separated species. Analyses of the genome-wide data showed that individuals group into three genotypic clusters. Cluster 1 (red) comprises populations from the Atlantic Forest province (ES, RJ) of the Parana dominion, representing *An*. *darlingi*. Cluster 2 (green) includes representatives from localities within the Parana Forest province of the Parana dominion (SP, MG, PR) with one Cerrado province population (Chacoan dominion). Cluster 3 (blue) incorporates the Boreal Brazilian and South Brazilian dominion populations (with one Cerrado province population) (Fig 1). Thus, the Cerrado province population is split between clusters 2 and 3. There is significant level of divergence between the Boreal Brazilian and South Brazilian dominion populations. (Amazonian populations) (Cluster 3) and the non-Amazonian populations (Clusters 1 and 2), but this divergence is only 50% of that seen between Clusters 1 and 2. Based on these findings, on low admixture between Clusters 1 and 2 (Fig 2), and on previous data demonstrating that a physical barrier, e.g., the Serra do Mar on the Atlantic coast, restricts gene flow between *An*. *darlingi* populations from the Atlantic Forest province and the remaining populations from the Chacoan and Brazilian subregions [13], we propose that Cluster 2 populations represent putative *An*. *paulistensis*. Within the western Atlantic forest, there is evidence from studies using multiple markers that the coastal mountain range limits dispersal in the bromeliad malaria vector complex *Anopheles* (*Kerteszia*) *cruzii*, such that different putative species have evolved [44, 45]. This finding lends support to our hypothesis of possible species-level differentiation between Clusters 1 (putative *An*. *darlingi*) and 2 (putative *An*. *paulistensis*).

Cluster 3 populations represent the Boreal Brazilian dominion (AM, AP) and South Brazilian dominion (AC, MT, PA, RO) both within the Brazilian subregion; in addition, this cluster includes individuals from the Cerrado province (TO) of the Chacoan dominion. There is a low level of allele sharing between clusters 2 and 3. One of these individuals is from Cerrado province (TO) population and the other sample is from Madeira province (MT) (Fig 2). The shared polymorphism of a second individual between Cerrado province (TO—cluster 3) and Parana Forest province (cluster 2) suggests that the former is a transition zone, with some attributes of both Amazon and West Atlantic Forest. A similar occurrence was observed in the population from Paraná province in the West Atlantic Forest (cluster 2), with one individual from PR sharing polymorphisms with the southeast cluster 1 (RJ, ES).

If our inference for *An*. *darlingi*, based on Morrone [22, 26] of possible speciation level divergence between Brazilian (cluster 3) and Chacoan subregions (clusters 1 plus 2), and between Atlantic Forest (cluster 1) and Parana Forest (cluster 2) provinces is accurate, other Neotropical organisms with similar distributions may be expected to show similar biogeographic or phylogoegraphic patterns. In fact, Costa [46], using data from the mitochondrial cytochrome *b* gene, observed that small forest-dwelling mammals distributed between and within the major forest domains of the Amazonia and Atlantic Forests and the intervening interior forest of Brazil diverged significantly. Between sister taxa of Neotropical orchard bees, Silva et al. [47] found that climatic oscillations that further separated these two large forest biomes promoted parapatric speciation, in which many species had their continuous distribution split, giving rise to different but related species. In the pantropical tree genus *Manikara*, the divergence between Atlantic coastal forest and Amazonian clades coincided with the formation of drier Cerrado and Caatinga habitats between them [48]. A clade of the frog *Hypsiboas albopunctatus* from the central Cerrado was found to have diverged from a southeastern clade (Brazilian Atlantic Forest) during the mid-Pleistocene [49]. Soil microbial acidobacteria 16S rRNA sequences are highly differentiated between Cerrado province (of Chacoan dominion) and Atlantic Forest (of Parana dominion), correlated with the distinctive soil and vegetation in each biome [50].

In addition, Nihei and Carvalho [51] defended the hypothesis that the vast Amazon region is not a biogeographical unit, but it is divided into southeastern and northwestern portions. The southeastern portion is closely related to the Chacoan and Parana dominions. These dominion relationships were inferred based on biogeographical patterns obtained for species of the genus *Polietina* (Diptera: Muscidae) from the Neotropical region. The fact that the *An*. *darlingi* population from Tocantins state (Cerrado province, Chacoan dominion) clustered with populations from the South Brazilian dominion may be a consequence of phylogenetic and biogeographical patterns that promoted the division of the forest biomes of the Neotropical region into the main components postulated by [52]. Consequently, two *An*. *darlingi* population of the Cerrado province (Chacoan dominion) did not cluster together but split into two clusters representative of the Brazilian dominion (cluster 3) and Parana plus Chacoan dominions (cluster 2). Alternatively, our results may be a consequence of sampling strategy with only two populations from the Chacoan dominion, which did not allow a clear separation among distinct biogeographical components postulated by Morrone [2, 6].

It is noteworthy that *An*. *darlingi* was described by Root [4] using specimens from a locality in Rio de Janeiro state (RJ) situated within the Atlantic Forest province (Fig 1), which clustered with representatives of ES, from the same province. In contrast, the MG, SP and PR populations from the Parana Forest province (with one Cerrado province population—SP) clustered separately. We hypothesize that the Parana Forest province cluster may represent the putative *An*. *paulistensis*, described by Galvão et al [5] from samples captured in Pereira Barreto, formerly Lussanvira municipality, in the West Atlantic Forest within the Parana Forest province. This species was synonymized with *An*. *darlingi* by Lane [6]; here we propose that *An*. *paulistensis* may be a valid putative species of the subgenus *Nyssorhynchus*. The genetic divergence between clusters 1 and 2 and the fact that cluster 3 is equally divergent from the other two clusters could also indicate that heterogeneous divergence among populations of *An*. *darlingi* was caused by ecological selection pressures and historical biogeographical processes that may have allowed the contact and separation among distinct populations during the historical events that had led to major Brazilian biome formation.

Several recent studies have led to the discovery of heterogeneous divergence across anopheline genomes under eco-environmental selection pressure [53–55]. Such investigations have provided details of population differentiation that contribute to a more precise understanding of mechanisms of divergence and speciation of particular interest to vector biology. This is amply demonstrated by critical evidence that the M (*An*. *coluzzii*) and S (*An*. *gambiae*) forms, recently described as valid species, continue to differentiate [56]. Further study into the genomic patterns of differentiation in *An*. *darlingi* may shed light on the mechanisms underlying its significant vectorial capacity in the Neotropics, and also help to clarify the vector status of the species in areas outside and inside the Amazon River basin.

## Supporting Information

S1 Fig STRUCTURE analysis of full SNP dataset with 11,533 loci. (PDF)

S1 Table Per-individual detail of the number of sequence reads and unique stacks genotyped. (XLSX)

S2 Table Population-level q values from STRUCTURE analysis. (CSV)

S1 Text Bash script with commands used to run the STACKS pipeline and STRUCTURE analysis. (TXT)
