# Supplementary material for: Brazilian Anopheles darlingi Root (Diptera: Culicidae) Clusters by Major Biogeographical Region
Source: PLoS One. 2015 Jul 14;10(7):e0130773. doi: 10.1371/journal.pone.0130773 (PMC4501553; doi:10.1371/journal.pone.0130773)
Supplement: S1 Fig — (PDF) [file pone.0130773.s001.pdf]

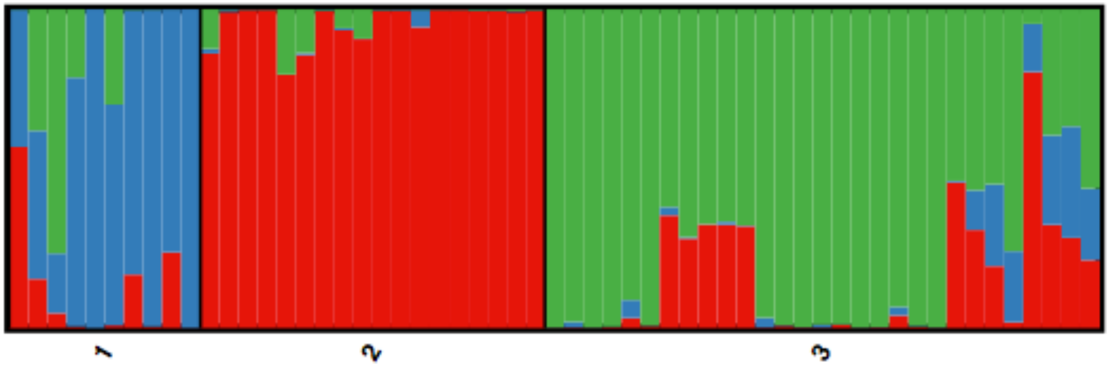


**S1 Fig. STRUCTURE analysis of full SNP dataset with 11,533 loci.** Initial analysis of the full dataset that was used to define the number of clusters broadly such that we were able to identify loci that were genotyped in > 75% of individuals within each cluster for fine-scale analysis of a more robust dataset where most of the genotypes were complete. STRUCTURE analysis was performed under the same parameters as outlined in the text for the filtered SNP dataset. In this figure, blue corresponds with cluster 1, red with cluster 2 and blue with cluster 3 from the text.
